# Food purchasing decisions of Malawian mothers with young children in households experiencing the nutrition transition

**DOI:** 10.1016/j.appet.2020.104855

**Published:** 2021-01-01

**Authors:** Valerie L. Flax, Chrissie Thakwalakwa, Courtney H. Schnefke, John C. Phuka, Lindsay M. Jaacks

**Affiliations:** a Public Health Research Division, RTI International, 3040 E. Cornwallis Road, Research Triangle Park, NC, 27709, USA; b Centre for Social Research, Chancellor College, University of Malawi, P.O. Box 280, Zomba, Malawi; c College of Medicine, University of Malawi, P/Bag 360, Chichiri, Blantyre, Malawi; d Department of Global Health and Population, Harvard T.H. Chan School of Public Health, 665 Huntington Avenue, Boston, MA, 02115, USA

**Keywords:** Overweight, Mother, Child, Food choices, Food purchasing decisions, Qualitative methods, Participant observation, Sub-Saharan Africa

## Abstract

As overweight/obesity prevalence increases in sub-Saharan Africa, information is needed about factors influencing food purchases in households with overweight members. This study assessed food purchasing decisions of Malawian mothers with young children (N = 54 dry season, N = 55 rainy season) among whom the mother, child, or both were overweight. Research assistants completed structured observations of mothers shopping for food during the dry season and of the types and quantities of foods in mothers' homes during the rainy season. After each observation, research assistants conducted an in-depth interview about factors that influenced food purchases, including asking mothers to sort 12 factors into piles that always, sometimes, or never influence their food purchases. Observations showed mothers most often shopped at outdoor markets to buy foods needed to prepare relish, such as tomatoes (71%), green leafy vegetables (58%), cooking oil (58%), and fish (40%). At home, maize flour (80%) and salt (66%) were the most common foods. Pile sorts and in-depth interviews revealed cost, taste preferences, freshness, and healthiness were the strongest factors influencing food purchases. Mothers described buying a smaller quantity or making substitutions (e.g., fish instead of meat) if a food is too expensive. Many mothers reported buying foods their family likes and prioritizing children's preferences. Freshness of foods, especially fruits and vegetables, and whether foods were perceived to be healthy also influenced food purchases, but mothers' knowledge of which foods were healthy was mixed. Mothers used some of their minimal funds to buy unhealthy foods (e.g., packaged or fried snacks) for their children, despite their overall emphasis on food cost and healthiness. These findings can be used by programs to reinforce healthy and decrease unhealthy food purchases by mothers with young children in Malawi.

## 1 Introduction

The prevalence of overweight and obesity among women and children in sub-Saharan Africa has steadily increased since 1975 (NCD Risk Factor Collaboration (NCD-RisC), 2017). While Malawi is among the countries with the lowest gross domestic product per capita globally (World Bank, 2019), it has not escaped the obesity epidemic. Forty-four percent of urban and 27% of rural women (Price et al., 2018) and 5% of children in both urban and rural areas are overweight or have obesity (National Statistical Office Malawi & ICF, 2017). In Malawi, higher wealth status, older age, ethnic group, and urban residence are associated with overweight/obesity among women (Mndala & Kudale, 2019), and male sex, higher household wealth status, older age, higher maternal body mass index (BMI), and maternal employment outside the home are associated overweight/obesity among children (Mtambo, Masangwi, & Kazembe, 2015; Ntenda, Mhone, & Nkoka, 2019). As Malawi and other sub-Saharan countries go through the nutrition transition, an increasing percentage of households have members with different nutritional status (e.g., an overweight mother with a normal weight or wasted child) (Popkin, Corvalan, & Grummer-Strawn, 2020).

Dietary intake is one of the most proximal factors leading to child and maternal nutritional status, including weight status (UNICEF, 1990). The acquisition and consumption of the food that makes up diets is influenced by factors within the external and personal domains of the food environment (Turner et al., 2018). The external domain includes elements of the food environment that are outside an individual's control, such as availability, price/cost, vendor and product properties, and marketing and regulation. The personal domain includes individual factors that influence food choice, such as accessibility, affordability, convenience, and desirability. The way these factors interact with each other and affect food choice is context dependent because it is embedded within specific food, sociocultural, and economic systems.

Several previous studies have investigated different aspects of the food environment and food choice in sub-Saharan Africa. A review of research on drivers of dietary behaviors among African women in urban settings found that in the external domain, factors such as quality/freshness of food, packaging, food prices, food availability, and media contributed to dietary choices (Gissing, Pradeilles, Osei-Kwasi, Cohen, & Holdsworth, 2017; Holdsworth & Landais, 2019). Type of food vendor, particularly the increasing presence of supermarkets in Africa, also influenced type and availability of food (Holdsworth & Landais, 2019; van Berkum, Achterbosch, & Linderhof, 2017). In the personal domain, taste preferences, hunger, mood, body image, perceptions of diet quantity or quality, knowledge of foods, food expenditures, convenience, and cultural beliefs were associated with dietary behaviors (Becquey, Savy, Danel, & et al., 2010; Charlton, Brewitt, & Bourne, 2004; Flax, Thakwalakwa, Phuka, & Jaacks, 2020; Gissing et al., 2017; Savy, Martin-Prevel, Danel, & et al., 2008; Waswa, 2011). A study in Cape Verde found that the top factors driving food choice in that context were food prices and perceptions that the food contributed to wellbeing, had good sensory appeal, was nutritious, and was natural (i.e., not processed) (Cabral, de Almeida, & Cunha, 2017). A study with mothers of young children in Madagascar found that health, child food preferences, cost, and food availability were the most important considerations during food shopping, whereas cleanliness and perception of vitamin and energy content of the food were most important when cooking (Farris, Misyak, O'Keefe, VanSicklin, & Porton, 2019). Cleanliness and nutritional content of the food were also key aspects for parents when selecting foods for their school-aged children in Madagascar (Rakotosamimanana, Arvisenet, & Valentin, 2014).

Previous studies to understand dietary behaviors and food choice in sub-Saharan Africa provide useful insight, but there are several important gaps that this study seeks to fill. First, previous research on food choice has mainly been quantitative. Because food choice is influenced by a wide variety of factors, including individual attitudes, beliefs, and preferences; cultural factors; market and product factors; and economic factors, it may be necessary to use both qualitative and quantitative methods to gain a deeper understanding. Second, few studies conducted in Africa have investigated food choice as it pertains to children or within households where mothers and/or children are overweight/obese. Third, previous studies have not measured whether food choice differs by season or urban/rural location. This research was part of a study of drivers of food choice in Malawian mothers with young children, among whom the mother, child, or both were overweight. Our aim was to use a mix of qualitative and quantitative methods during the dry and rainy seasons to better understand the factors involved in food purchasing decisions among urban and rural women with young children in Malawi whose families are experiencing the nutrition transition.

## 2 Methods

### 2.1 Study location and design

This study was conducted in Lilongwe and Kasungu Districts in central Malawi. These areas were chosen because our focus was on households in which either the mother, the child, or both were overweight or had obesity and central Malawi has the highest prevalence of overweight and obesity in the country (National Statistical Office Malawi & ICF, 2017). To better understand if there were urban/rural differences in food choice, we purposefully selected two urban neighborhoods and two rural villages in each district.

This analysis is part of a larger study of drivers of food choice among mothers with young children in Malawi. For the component of the study described here, we used three methods with the same participants: structured direct observations, in-depth interviews, and pile sort exercises (Bentley, Boot, Gittelsohn, & Stallings, 1994; Pelto, Armar-Klemesu, Siekmann, & Schofield, 2013). We selected these methods to obtain rich data and descriptions of factors that influence food purchasing decisions. We collected data during the dry and rainy seasons to determine if seasonal variations in food availability and prices influence food choice.

### 2.2 Study population

For the larger study, community leaders in each location invited adult women with children 6–59 months of age for anthropometric screening using standard techniques (Cogill, 2003). They were purposefully enrolled in three study groups based on standard anthropometric cutoffs (World Health Organization, 2009). Mother-child pairs were eligible to participate in the study if both the mother (BMI ≥ 25 kg/m^2^) and child (WHZ > +2 SD) were overweight/obese; the mother was overweight/obese and the child was normal weight (−2 SD < WHZ ≤ +2 SD); or the mother was normal weight (18.5 kg/m^2^ ≤ BMI < 25 kg/m^2^) and the child was overweight/obese.

For the subsample included in observation, in-depth interview, and pile sort data collection, research assistants selected participants proportional to the size of the study group in urban and rural areas in each district during the dry season. They revisited the same women to collect data in the rainy season and replaced those who had moved or refused to be interviewed a second time with another woman in the same study group and urban/rural category.

### 2.3 Data collection

Six research assistants with previous experience collecting nutrition data participated in a five-day training prior to screening participants and collecting data during the dry season. They participated in a three-day refresher training before starting rainy season data collection. Both trainings included pilots of data collection procedures. Data collection tools were developed in English and translated into Chichewa and are included in Supplemental Materials. Participants received an incentive of four US dollars for each study visit. Data collection visits in each season started with an observation. During the dry season, research assistants made an appointment with each mother to accompany her on a food shopping trip. Mothers were instructed to consider this a regular food shopping trip and to go to the markets or shops they would usually visit to obtain the items they wanted to purchase that day. The study team did not provide money for shopping to participants. Research assistants recorded information on a structured observation form about food purchases and observable drivers of food choice during the shopping trip, including type and quantity of foods purchased, location of purchase, cost of each item, and factors that influenced food choices (freshness, availability, price, other). Research assistants documented observable factors based on mothers' interactions and conversations with vendors; questions about these factors or drivers of food choice were reserved for the interview after the observation. After conducting and analyzing the market trip observations, we found that the foods observed were mainly perishable items. To capture a wider variety of foods, including staples and dry goods, during the rainy season, research assistants observed foods available at mothers’ homes. They used a structured observation form to document foods in the homes, quantity of each food, whether it was purchased or self-produced, time since purchase, frequency of purchase, and monetary value of the item. Observation data were collected on paper data collection forms and entered into Excel spreadsheets for analysis.

Following the observation in each season, research assistants conducted an in-depth interview and pile sort exercise with the mothers. Most of the questions during both seasons were the same. Topics included: main influencers of where food is purchased; main factors that most and least influence types of food purchased; and the influence of food availability, cultural beliefs about foods, taste preferences, cost, and time constraints/convenience on food purchasing decisions. Both question guides asked about specific foods purchased for the child. We added a question in the rainy season about specific foods the mother purchases for herself. The dry season question guide also included questions about the specific foods purchased during the market trip, while the rainy season question guide asked a series of questions about the foods that were in the household. In-depth interviews were digitally recorded, with the participant's permission. Recordings were transcribed verbatim in Chichewa, then translated into English.

The pile sort was conducted at the end of the in-depth interview and was the same during both seasons. Participants were asked to sort 12 factors: hunger/appetite, taste, food safety, marketing/advertisements, mood, perceived healthiness of the food, cooking skills, foods linked to health status (e.g., foods eaten when ill), cost of food, seasonal availability, time available for preparation, and attitudes/beliefs about specific foods (Bailey et al., 2018). The factors were printed in Chichewa on separate pieces of cardstock and laminated. Research assistants mixed the cards, read them aloud one by one in a random order, and asked the women whether it always, sometimes, or never influences their own food choices. Research assistants wrote the results of the pile sort on the paper question guide for each participant. The data was entered into an Excel file for analysis.

### 2.4 Data analysis

We calculated descriptive statistics for participant characteristics and for observation and pile sort data using Stata MP (version 16.0, StataCorp, College Station, Texas). We created an asset score based on presence/absence of 12 household assets and resources (electricity, koloboyi (type of lamp), paraffin lamp, radio, television, mattress, sofa, table and chairs, refrigerator, bicycle, watch, mobile phone) included in the Malawi Demographic and Health Survey (National Statistical Office Malawi & ICF, 2017) and calculated the household food insecurity access score (HFIAS) as indicators of household socioeconomic status (Coates, Swindale, & Bilinksy, 2007).

We coded and analyzed the in-depth interviews using qualitative content analysis methods (Hsieh & Shannon, 2005). We developed codebooks of deductive codes separately for each season. Two research assistants in Malawi coded the transcripts in Dedoose (versions 8.1.8. and 8.2.14., SocioCultural Research Consultants, LLC, Los Angeles, California). We ensured intercoder reliability by checking 10% of their coding against an investigator's coding. We then created data matrices for the analysis with themes in the columns and rows for each participant (Miles & Huberman, 1994). The research assistants used code reports to populate the matrices with illustrative quotes.

We analyzed observation data by study group (i.e., overweight mother with overweight child, overweight mother with normal weight child, and normal weight mother with overweight child) and urban/rural location but did not compare seasons because the data collected were different. We tested differences in pile sort rankings by season and within each season by study group and location using Fisher's exact tests. For interview data, we analyzed differences in responses or emphasis by study group, urban/rural location, and season.

## 3 Results

The final sample for this analysis included 54 mothers during the dry season and 55 during the rainy season (see Supplemental Table 1 for percentage of sample by study group). Thirty-five mothers participated in both seasons. We added 20 new participants in the rainy season because participants had moved and could not be contacted again or refused to participate. Dry season participants who were not interviewed during the rainy season were more often mothers who lived in urban areas and had more education than those who stayed in the sample (Supplemental Table 2). Characteristics of participants in the dry and rainy seasons were similar (Table 1), in part because more than half of the participants were the same individuals. On average, mothers in our sample were 29 years old and their children were 2 years old. Less than 40% of the mothers had at least some secondary education. Although the households had few assets on average, their mean HFIAS was low, indicating most households were food secure. By design, approximately half of our sample lived in urban areas.

**Table 1:** Participant characteristics.

Characteristics	Dry season (N = 54)	Rainy season (N = 55)
Mother's age, years, mean (SD)	29.3 (8.0)	29.2 (7.3)
Child's age, months, mean (SD)	26.7 (15.8)	24.8 (14.4)
Mother's has some secondary education or above, N (%)	20 (37%)	21 (38%)
Household Food Insecurity Access Score (range 0–27), mean (SD)	4.2 (5.6)	6.1 (6.5)
Household assets (range 0–12), mean (SD)	3.6 (2.7)	4.8 (3.1)
Household is in urban location, N (%)	25 (46%)	27 (49%)

We did not find differences by study group for the observations or in-depth interviews. Therefore, we present the combined results for our three study groups for those parts of the analysis.

### 3.1 Observations

During the market trips conducted in the dry season, overall, 83% of mothers shopped only at outdoor markets, 9% shopped at outdoor markets and shops/groceries, and 8% shopped only at shops/groceries. More mothers in urban areas (24%) bought at least some items at shops/groceries than in rural areas (13%), but most food in both locations was purchased in outdoor markets. Mothers mainly purchased food to make relish, a dish to accompany stiff maize porridge (*nsima*), the staple food. Overall, the most frequently purchased items were tomatoes (71%), green leafy vegetables (58%), cooking oil (58%), fish (40%), onions (37%), meat (15%), eggs (10%), groundnut flour (10%), and beans (10%). The top five items were the same in urban and rural areas, although their relative order was slightly different [tomatoes (urban 65%, rural 76%), green leafy vegetables (urban 61%, rural 55%), cooking oil (urban 52%, rural 62%), fish (urban 52%, rural 31%), onions (urban 39%, rural 34%)]. Overall, other foods purchased that were not for main meals were fruit (21%), sweets (biscuits, hard candies, and freezes/popsicles; 13%), Kamba puffs (a packaged processed maize snack; 12%), fried snacks (doughnuts, banana fritters; 8%), and sugar-sweetened drinks (fizzy drinks (sodas) and sweetened grain drinks; 6%). Mothers in urban areas purchased fruit more often than those in rural areas (30% vs. 14%), and women in rural areas purchased fried snacks (14%), while women in urban areas were not observed buying them. Mothers purchased an average ± SD of 4 ± 2 food items each, which did not differ by urban/rural location. Research assistants observed drivers for the selection of 75% (165/219) of the items purchased. Mothers selected 42% of the foods for their freshness, 34% for their price, and 25% for seasonal availability; there was no difference in observed drivers of food selection by location. Overall, the mothers’ median (IQR) expenditure on all food items was 635 (290, 1100) Malawi Kwacha (1 USD = 720 MK). The median expenditure on observed food purchases was higher in urban [760 (500, 1400) MK] than rural areas [500 (250, 1000) MK]. Sixty percent of mothers bought foods specially for their young child during the market trip.

During the rainy season, an average ± SD of 5 ± 2 food items were observed in households overall, with a slightly lower average in rural than urban areas. Overall, the most common food items in the households were maize flour (80%); salt (66%); sugar (39%); cooking oil (34%); tomatoes (30%); green maize (30%); beans (18%); green leafy vegetables, onions, and bread (14%); and pumpkins and Irish potatoes (11%). The top five items were the same in urban and rural areas, although the percentages differed by location for some items [maize flour (urban 72%, rural 89%), salt (urban 48%, rural 85%), sugar (urban 41%, rural 37%), cooking oil (urban 45%, rural 22%), tomatoes (urban 31%, rural 30%)]. A variety of other foods, such as sweet potatoes, fish, groundnuts, soya, eggs, Kamba puffs, and fizzy drinks, were logged in fewer households (<10% each). Among households with each of the following food items, the median (IQR) quantity recorded was 10 (2, 20) kg of maize flour, 250 (130, 500) g of salt, 500 (250, 1000) g of sugar, and 208 (61, 1000) ml of cooking oil. Households had similar quantities of maize flour in urban and rural areas, but urban households had larger quantities of salt, sugar, and cooking oil on hand than rural households with these items. Of the 281 food items logged across households, 90% were purchased (urban 93%, rural 85%) and 10% were self-produced (urban 7%, rural 15%). Overall, the median (IQR) value of the foods in each household was 5000 (1805, 9035) Malawi Kwacha. The median (IQR) value of foods in the household was higher in urban [7400 (1950, 11300) MK] than rural [3910 (1750, 6920) MK] areas. Foods such as maize flour and salt were usually purchased weekly or monthly. Foods such as sugar and cooking oil were purchased daily or weekly. Other, more perishable foods, such as tomatoes and green leafy vegetables, were purchased daily or every few days.

### 3.2 Pile sorts

**Fig. 1:**
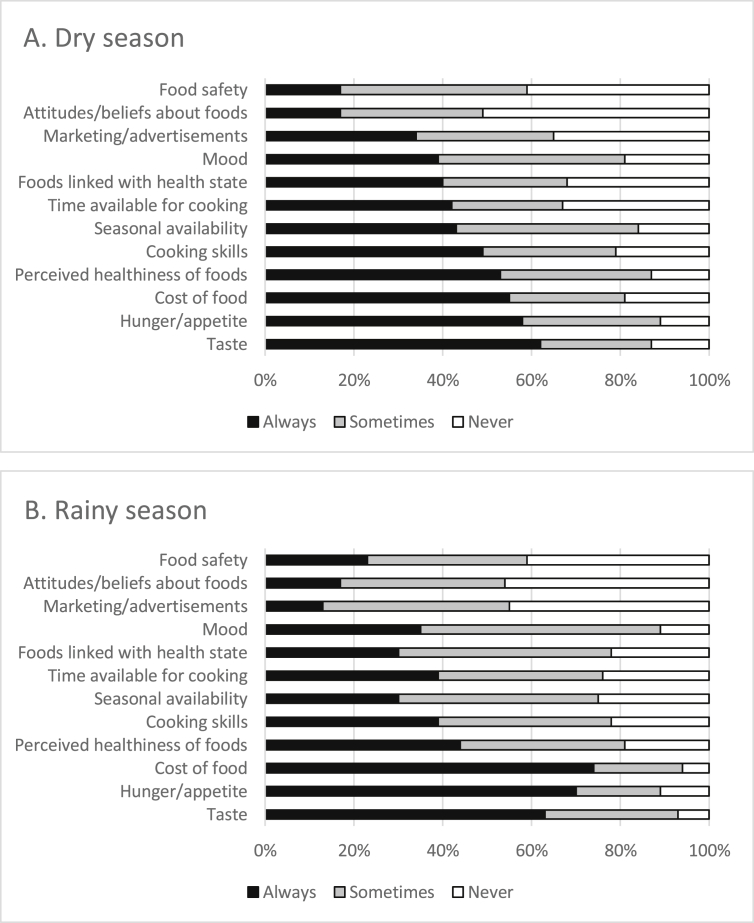
A and B: Influence of 12 drivers of food choice on food purchasing decisions during the dry and rainy seasons.

Overall, during the dry season, the drivers that women most often said “always” influence their food choices were taste (62%), hunger/appetite (58%), cost of food (55%), and perceived healthiness of the food (53%) (Fig. 1A). During the rainy season, cost (74%), hunger/appetite (70%), and taste (63%), and perceived healthiness of the food (44%) remained the most frequent drivers that always influence women's food choice, but their order and relative importance shifted (Fig. 1B). Cost was the only factor that trended toward a difference by season (55% dry, 74% rainy; p = 0.05). Food safety, attitudes/beliefs about foods, and marketing/advertisements were the three least influential drivers during both seasons. Among the common drivers, we found no differences by study group in the dry season and the only difference in the rainy season was for hunger/appetite. Normal weight mothers more frequently reported that hunger/appetite always influenced their food choices than overweight mothers (83% normal weight mother, overweight child; 67% overweight mother, normal weight child; 61% overweight mother, overweight child; p = 0.03).

Drivers of food choice by season broken down by urban/rural location are shown in Supplemental Fig. 1. In the dry season, taste, perceived healthiness, cost, seasonal availability and hunger/appetite were the most prominent drivers of food choice in urban areas and hunger/appetite, taste, cost, cooking skills, and perceived healthiness were the most common in rural areas. In the rainy season, cost, hunger/appetite, and taste were the most important drivers in both urban and rural areas. We did not find any significant differences in drivers of food choice by urban/rural location within either season.

### 3.3 In-depth interviews

The key themes that emerged from the in-depth interviews were related to specific drivers of food choice or food purchasing decisions and were mainly the same in both seasons. In general, we did not find obvious patterns by urban/rural location, but we note when a strong pattern emerged with respect to specific themes. Cost of food, taste preferences, need for specific ingredients for meals, quality in terms of freshness/appearance of food, and perceived healthiness of food were mentioned during interviews as the most important factors influencing food purchases. Mothers also described the foods they buy for their young children and themselves and the drivers of those types of food purchases.

#### 3.3.1 Drivers of household food purchases

Cost of food and subsequent affordability were spontaneously named as drivers by more mothers during the rainy than dry season, which aligns with the findings from the pile sorts. Mothers in both urban and rural areas described cost as influencing the types of food they buy. For many, their food choices were constrained by their financial situation. A mother in a rural area during the rainy season (ID 347) explained, “*Mostly [my food choices are] influenced by the amount of cash at hand. This factor determines what type of food I buy.*” Another mother gave an example of the types of foods she would buy if she had only a little money available and what she would add if she had more funds.

“*It is based on the money you have. For example, you buy mustard greens and tomatoes, then you go cook. When you have enough money, you buy mustard greens, tomato, onion, even cooking oil, then you go cook for the family. And another day, if you have money, you add more on the list, like milk and sweet potatoes…But we cannot afford all of these for we do not have enough resources.*” Mother in a rural area during the dry season (ID 212)

The cost of food in relation to the amount of money a mother had available also affected the quantity of food she was able to buy. A mother in an urban area during the rainy season (ID 30) said, “*I consider the money at hand in relation to the quantity that I need. I have to buy the food that [is enough for] the whole family to eat.*” If a mother does not have enough money to buy the quantity of food she needs to feed the family, she selects another type of food or buys less. For example, a mother in a rural area during the dry season (ID 64) explained, “*Pork is a bit cheaper…if you compare it with goat meat…This amount of pork meat is enough for two meals for my family, while the same amount of goat meat would not be enough for two meals.*” A mother in an urban area during the dry season (ID 320) who discussed buying less food in accordance with the cost and the funds available said, “*It varies depending on the cost. Sometimes I buy four [tomatoes] at 100 Malawi Kwacha. On other days, when they go up, I just buy two.*” Some women described buying in bulk to save money and for convenience. A mother in an urban area during the rainy season (ID 60) explained, “*I buy enough cooking oil for the whole month. I see it as cheaper that way.*” For perishable foods, avoiding waste by buying an appropriate amount that could be consumed in one day was also a consideration. A mother in an urban area during the dry season (ID 136) said, “*I buy one bunch [of green leafy vegetables] because I don't want them to get bad. I want to buy what we can eat in one day.*”

Taste of food or the family's food preferences was important for mothers' food purchasing decisions during both seasons. Many mothers said they specifically buy foods that they know their family likes to eat and that will make their family members happy. Sometimes they considered their own preferences, but they often described putting other family members' preferences ahead of their own. A mother in an urban area during the dry season (ID 320) said, “*In a family, it is very important to make everyone happy. Sometimes we just buy food because the children like it, and we have to suppress how we feel about it.*” Some mothers buy foods to cater to the tastes of their children or to ensure that food is not wasted because no one in the household wants to eat it. A mother in a rural area during the rainy season (ID 324) explained, “*I have noticed that pumpkins are the only food that when I give to my children in the morning they eat happily and get full compared to things like bread.*”

Mothers mentioned that the need for specific ingredients or specific types of food used to prepare daily meals drove their food purchases. These ingredients and combinations of foods are influenced by the Malawian food culture, in which the midday and evening meals are comprised of the staple food *nsima* accompanied by one or more side dishes of relish, often made of fish, green leafy vegetables, or beans with tomatoes and onions. Many of the mothers’ comments were related to buying ingredients for relish. For example, the interviewer asked a mother in a rural area during the dry season (ID 93) about the most important factor she considers when buying food and she replied, “*Relish. We need relish [including] salt and tomatoes [to eat with]* nsima.” A mother in an urban area during the dry season (ID 267) also explained, “*Mostly I think of relish because in most cases [maize] flour [for* nsima*] is always available.*”

Quality of food was not included as an item in our pile sorts but was spontaneously named by mothers as one of the top factors that drives their food purchases. For women in this study, quality was related to the appearance and freshness of the food and was not equated with food safety. They mentioned quality in relation to all types of food but especially with respect to vegetables, fruits, and fish. Food quality was mentioned by more women as a driver of food purchases during the dry than the rainy season and by more normal weight mothers with overweight children in both seasons. Many mothers gave general statements about the importance of food quality. A mother in a rural area during the dry season (ID 282) said, “*I consider the appearance of the food item. It has to be of good quality.*” Mothers also described how they would check foods for signs of poor quality so they could find foods that they perceived as having higher quality. For example, a mother in a rural area during the dry season (ID 196) said, “*The appearance of the food… The way it looks. If it has any problems, like the tomatoes, we see if it has any holes.*” Some of the mothers also explained how they bought certain foods in specific types of markets or with vendors who they perceived to have higher quality food. For example, a mother in an urban area during the dry season (ID 35) explained after the market observation, “*In Chipiku [grocery], you are assured of quality. Cent Market [outdoor market] is where I buy my relish, while tangerines I bought from that boy because he had quality tangerines.*”

Perceived healthiness of the food was an important factor driving food purchasing decisions but was mentioned less frequently during both seasons than factors like cost, taste, and quality of the food. Mothers who discussed the healthiness of foods want to buy foods that they perceive are nutritious and will make their family, and especially their children, healthy and strong. A mother in an urban area during the dry season (ID 136) said, “*Apart from price, I think of what the food is going to do in the body.*” A mother in an urban area during the rainy season (ID 169) explained, “*I mean everything I am considering when buying food is important. I do consider health boosting foods, such as milk, margarine, and biscuits.*” The types of foods that women perceived as healthy varied widely, ranging from green leafy vegetables, fruits, beans, and whole grain maize flour to fizzy drinks (or sodas), biscuits, and margarine. For example, a mother in a rural area during the rainy season (ID 118) explained, “*Oranges, bananas, and beans [are considered good]. People say these foods are nutritious, good for children's health.*” Fizzy drinks and sweetened grain drinks were described by a few mothers as increasing blood or making people strong.

#### 3.3.2 Drivers of food purchases for young children

During dry season interviews, mothers said the food they purchase specifically for their child are sweets, biscuits, sugar cane, freezes, Kamba puffs, fruit, and fried snacks, which aligns with the findings from our observations. During rainy season interviews, mothers mentioned buying Kamba puffs, fizzy drinks, sweets, biscuits, sugar cane, freezes, *maheu* (sweetened grain drink), *thobwa* (fermented maize drink), milk, yoggie (sweetened liquid yogurt), fried snacks, and fruit. During both seasons at least three-quarters of the mothers said they buy foods specifically for their child daily or regularly; mothers in urban areas more often said they buy them daily than those in rural areas. The top reasons mothers gave for buying these food items were the same during both seasons and in urban/rural locations, including because the child likes them, the mother wants the child to feel loved and be happy, the mother considers the food to be healthy and wants the child to be healthy, as a snack to keep the child from getting hungry, and as a way to appease a misbehaving or crying child. Illustrative quotations for each reason are shown in Table 2. Many of the quotations include two or more of these reasons for buying the child specific foods.

**Table 2:** Reasons mothers buy foods specifically for their young children.

Reasons for buying specific foods for child	Illustrative quotations
Child likes the food item	*“Freezes [popsicles], Kamba puffs [processed maize snacks], Lays potato chips, and sweets… We buy them because he enjoys eating them.”* Mother in urban area during rainy season (ID 31)
Mother wants the child to feel loved and be happy	*“She is the only child. I do buy her yoghurt, Kamba, juice, and sometimes Sobo [locally-produced fizzy drink]. I do buy them because she loves them and as a mother, I want my child to look healthy and feel loved. I do buy [them] regularly.”* Mother in urban area during dry season (ID 35)
Mother considers the food to be healthy and wants the child to be healthy	*“I [bought]* maheu*, milk, Kamba, and eggs…I buy them for the child to eat while he is young, for him to grow healthy.”* Mother in rural area during dry season (ID 212)
As a snack to keep the child from getting hungry	*“I bought fruits. This is so that they have food between meals to avoid going hungry.”* Mother in rural area during dry season (ID 84)
To appease a misbehaving or crying child	*“I buy Sobo… I also buy yoggie, Enjoy [fizzy drink], and Kamba puffs. I buy these foods so that I can be giving her when she is troubling [me]. I buy almost every day.”* Mother in urban area during rainy season (ID 60)

#### 3.3.3 Drivers of mothers’ food purchases for themselves

Mothers were asked about food purchases for themselves only during the rainy season. Nearly 90% said they buy foods specifically for themselves and about 50% said they buy them daily or regularly. The most common items in both urban and rural areas were fizzy drinks, fried snacks, fruit, sweets, biscuits, sugar cane, freezes, African cake, and cereals, grains, and tubers. Mothers in urban areas also frequently bought *thobwa* or *maheu* for themselves, while these items were not mentioned by mothers in rural areas. The most common reasons mothers gave for buying foods for themselves were: because they like them or have a craving for them, to satisfy hunger, and the food is perceived as having specific health benefits. Illustrative quotations for each reason are shown in Table 3.

**Table 3:** Reasons mothers buy foods specifically for themselves.^a^.

Reasons for buying specific foods for themselves	Illustrative quotations
They like them or have a craving for them	*“Sometimes you find that you've already eaten here at home and you're walking around and you just see something you have been craving so you end up buying it.”* Mother in rural area (ID 324)
To satisfy hunger	*“Because I can be somewhere without lunch, so the Fanta goes as a substitute.”* Mother in urban area (ID 169)
The food is perceived as having health benefits	Interviewer: “*Why do you buy avocado pears?”* Participant: *“I heard that they are helpful in blood production.”* Mother in rural area (ID 89)

a Data on mothers' purchases for themselves was collected only during the rainy season.

## 4 Discussion

In this study, taste preferences and the related desire for specific ingredients for relish, cost, quality or freshness of the food, and perceived healthiness of the food were the main drivers of food choice in this context. Although some factors, particularly cost, varied by season, we found no difference in urban/rural drivers of food choice in either season and minimal differences by study group. Overall, our different data collection methods converged toward a few key factors that influence mothers' food purchasing decisions across seasons in urban and rural areas of central Malawi. Many of the factors that emerged from our analysis fit in the desirability component of the personal domain of Turner's food environment framework, which includes preferences, acceptability, tastes, desires, attitudes, culture, and knowledge (Turner et al., 2018). Cost is also part of Turner's framework in the external domain as is affordability or purchasing power, which is part of the personal domain.

Cost was a major driver of food choice in our study and many participants appeared to be price sensitive. Our qualitative data showed that they shifted to less expensive foods or bought smaller quantities when prices increased because the item or quantity was no longer affordable. Similar findings have been reported in a recent quantitative analysis in Malawi (Pauw, Verduzco-Gallo, & Ecker, 2018) and in other food choice studies in low- and middle-income countries (Cabral et al., 2017; Daivadanam, Wahlstrom, Thankappan, & Sundari Ravindram, 2015; Farris et al., 2019). In our study, both the pile sorts and interviews showed that cost was more prominent during the rainy season, most likely because the price of maize, the staple food, is higher at that time of year. Although half of our sample was rural, most food items were purchased, not self-produced, most likely because these data were collected during the rainy season, when rural farmers have low stocks of self-produced food. Rural households also have less cash during the rainy season when they are waiting for the harvest and food prices are high, which explains why households in rural areas were observed spending less on food purchases and the value of the food in their homes was lower than in urban areas. Despite these differences, the total value of food items in homes and observed food expenditures was low in both areas, indicating that these families with overweight members had limited incomes. This aligns with evidence that overweight is increasing fastest in poor households in some other African countries (Ziraba, Fotso, & Ochako, 2009).

Shared cultural norms about which foods are appropriate for different meals and for people of different ages play a role in determining the foods that are available in markets and that people grow, purchase, and prefer to eat (Mintz & Du Bois, 2002). In Malawi, a strong norm persists for eating *nsima* accompanied by relish for the midday and evening meals. In our study, most households did not have refrigeration, even in urban areas, so women tended to focus on the perishable components of relish, such as tomatoes or green leafy vegetables, when they did their food shopping daily or every few days. Some mothers in our study also thought about *nsima* when they shopped, but as our observations showed many have some maize flour on hand and did not need to buy it every day. Buying ingredients for relish or *nsima* is driven, in part, by the normative map of foods and food combinations that are expected for different meals. This finding is similar to other drivers of food choice studies in low- and middle-income countries where habit and cultural perceptions were important drivers (Bailey et al., 2018; Cabral et al., 2017; Daivadanam et al., 2015). The pervasive food culture and shared cultural norms related to food in Malawi, with an emphasis on meals of *nsima* and relish, may also explain why we found few differences in drivers of food purchasing decisions between urban and rural areas or study group.

Taste preferences are also linked, in part, to the same food-related cultural norms and habits. We found that children's taste preferences sometimes take precedence to limit food waste in this setting where household budgets are quite limited. Similar findings have been reported in the U.S., where low-income mothers limited purchases of fruits and vegetables because they are perishable and their children do not like to eat them (Wiig & Smith, 2008).

Eating and giving their children foods they considered to be healthy or nutritious was important to mothers, and was also reported in two studies of food choice among mothers/parents of children in Madagascar (Farris et al., 2019; Rakotosamimanana et al., 2014). In our study, mothers’ knowledge of which types of foods are healthy was variable. They knew that vegetables and fruits are healthy, and more mothers in urban areas said they buy fruits specifically for their child, but some mothers also considered foods such as Kamba puffs, margarine, and fizzy drinks to be healthy or contain important nutrients. Although mothers considered the perceived healthiness of snack foods for themselves and their children, the drivers for these types of food purchases tended to be more emotional. For example, mothers said they buy snack foods, like Kamba puffs, sweetened yogurt, and fried foods, for their children to make them happy or appease them when they are crying and for themselves because they crave the food. Studies in other low and middle-income countries have shown that while mothers or parents say they want to feed their children healthy foods, they do not know that some of the snacks they give their children, especially those that are commercially produced, are not healthy (Pries et al., 2017; Sharma et al., 2019).

Food safety and convenience are two drivers of food choice that have emerged as key factors in other studies in low- and middle-income countries (Bailey et al., 2018; Daivadanam et al., 2015; Farris et al., 2019; Rakotosamimanana et al., 2014), but were not important in our study. The mothers in our study looked for fruits and vegetables that were fresh but did not otherwise mention cleanliness or food safety, and food safety ranked as one of the least important factors in our pile sorts. Most of our participants were not employed outside the home, they purchased raw foods/ingredients from outdoor markets for preparation at home, and ready-made meals are not widely available. This may explain why convenience was a less important driver of food choice in this study. Future research should explore perceptions of food safety and consumer trust in different cultural settings to better understand how these perceptions vary across contexts and how they can be used in behavior change communication strategies to promote consumption of fresh fruits and vegetables.

This study had some limitations. The observations were scheduled rather than unannounced, so mothers had an opportunity to plan for the visit, which may have influenced the observed behaviors. For the pile sort data, the replacement of mothers who were lost to follow up between seasons should be taken into consideration in assessing seasonal differences in drivers of food choice. In conducting the pile sorts using the same factors as Bailey et al. (2018), we found that some factors, particularly the link between a person's health status and eating certain types of foods, were difficult for participants to understand. In addition, some factors appear to be more important for decision-making about purchasing specific foods and not for others (e.g., freshness is important for vegetables, but not for grains). Future research on food purchasing decisions and food choice should try pile sorts for different food groups (e.g., fruits, vegetables, grains, meat).

In conclusion, we found that taste, cost, freshness, and healthiness were the main drivers of food purchasing decisions among Malawian mothers with young children in households affected by the nutrition transition. We identified a few leverage points for influencing healthy food consumption. Most of the households we studied purchased many of their food items rather than self-producing, which suggests that influencing their food purchasing choices through food pricing policies, food subsidies, and social behavior change communication could play an important role in shaping their diet by encouraging or discouraging purchase of certain foods. The Ministry of Health and its implementing partners could educate community members about which foods are healthy and unhealthy and build on the government's existing nutrition messages to encourage spending of limited household resources on a variety of different types of foods and on healthy foods, like fruits, vegetables, and eggs, rather than on unhealthy snacks, such as Kamba puffs, fried foods, sweets, or fizzy drinks (Government of Malawi & Scaling Up Nutrition, 2013). Targeting misperceptions around the healthfulness of prepared snack foods for children should be a priority as should the development of healthy, non-perishable snack foods.

## Source of funding

This research was funded by the Drivers of Food Choice (DFC) Competitive Grants Programs, which is funded by the UK Government's 10.13039/501100002992Department for International Development and the 10.13039/100000865Bill & Melinda Gates Foundation, and managed by the University of South Carolina, Arnold School of Public Health, USA. The views expressed do not necessarily reflect the UK Government's official policies.

## Contributor statement

VLF, CT, and LMJ designed the study. CT and JCP were responsible for data collection. CHS led the analysis of qualitative transcripts. VLF analyzed the observation and pile sort data and drafted the manuscript. All authors contributed to the final version of the manuscript.

## Ethics statement

This study was approved by the College of Medicine Research Ethics Committee at the University of Malawi and by the institutional review boards at RTI International and the Harvard T. H. Chan School of Public Health.

## Declaration of competing interest

The authors have no conflicts of interest.
