# Supplementary material for: Food purchasing decisions of Malawian mothers with young children in households experiencing the nutrition transition
Source: Appetite. 2021 Jan 1;156:104855. doi: 10.1016/j.appet.2020.104855 (PMC7677890; doi:10.1016/j.appet.2020.104855)
Supplement: Multimedia component 2 [file mmc2.docx]

**Supplemental Figure 1: Influence of 12 drivers of food choice on food purchasing decisions during the dry and rainy seasons in urban and rural areas**
