# Supplementary material for: Food purchasing decisions of Malawian mothers with young children in households experiencing the nutrition transition
Source: Appetite. 2021 Jan 1;156:104855. doi: 10.1016/j.appet.2020.104855 (PMC7677890; doi:10.1016/j.appet.2020.104855)
Supplement: Multimedia component 3 [file mmc3.docx]

**Supplemental Table 1: Participants’ study groups by season**

| **Study Group** | **Dry season**  **(N=54)**  N (%) | **Rainy season**  **(N=55)**  N (%) |
| --- | --- | --- |
| Overweight mother, overweight child  Overweight mother, normal weight child  Normal weight mother, overweight child | 15 (28%)  24 (44%)  15 (28%) | 18 (33%)  19 (34%)  18 (33%) |
|  |  |  |
