# Supplementary material for: Food purchasing decisions of Malawian mothers with young children in households experiencing the nutrition transition
Source: Appetite. 2021 Jan 1;156:104855. doi: 10.1016/j.appet.2020.104855 (PMC7677890; doi:10.1016/j.appet.2020.104855)
Supplement: Multimedia component 4 [file mmc4.docx]

**Supplemental Table 2: Characteristics of mother-child pairs enrolled in the dry season who remained in the sample during the rainy season and those who were not interviewed in the rainy season**

| **Characteristics** | **Remained in sample**  **(N=35)** | **Not interviewed**  **(N=19)** |
| --- | --- | --- |
| Mother’s age, years, mean (SD) | 29.8 (8.0) | 28.4 (8.1) |
| Child’s age, months, mean (SD) | 25.2 (15.0) | 29.4 (17.1) |
| Mother has some secondary education or above | 11 (31%) | 9 (47%) |
| Household Food Insecurity Access Score (range 0-27), mean (SD) | 3.7 (4.8) | 5.3 (6.7) |
| Household assets (range 0-12), mean (SD) | 3.4 (2.6) | 3.8 (2.8) |
| Household is in urban location | 14 (40%) | 11 (58%) |
