# Supplementary material for: Food purchasing decisions of Malawian mothers with young children in households experiencing the nutrition transition
Source: Appetite. 2021 Jan 1;156:104855. doi: 10.1016/j.appet.2020.104855 (PMC7677890; doi:10.1016/j.appet.2020.104855)
Supplement: Multimedia component 5 [file mmc5.docx]

**Food Preferences and Purchasing In-Depth Interview Guide**

Thank you for letting me ask about the foods you have available in your household. I’d now like to ask you some questions about foods you have at home and foods you purchase.

OPENING QUESTIONS

1. In general, when you go to buy food for your family, what are the main factors that influence where you buy the food?
2. In general, when you go out to buy food for your family, what are the main factors that influence what you buy?

Probes:

- - - What is the MOST important factor when you make food buying decisions?
    - What is the LEAST important factor when you make food buying decisions?

SPECIFIC FOODS & LOCATIONS

1. [Interviewer: Go through the list of food items in the household one-by-one and ask the questions below about each food.] I saw you have *(insert specific type of food)* in your house. What was it about that particular food that led you to buy it or to keep it on hand [if it is self-produced]?

Probes:

- - - How often do you have this food available in your household?
    - How often do you buy this food?
    - How much do you usually have on hand?
    - How much do you usually buy? What are the reasons for that?
    - Where do you usually buy it? (probe: open market, shop, grocery, street vendor)
    - How much do you sell or use to prepare food that you sell?

1. We have talked a lot about the foods you have in your house right now. I’d like to ask you about foods you do not have in the house now, but that you buy daily or weekly.
   1. What types of foods do you buy daily? Where do you buy them?
   2. What types of foods do you buy weekly? Where do you buy them?

SPECIFIC FACTORS

1. What types of foods or drinks do you buy specifically for yourself? What are your reasons for buying these foods or drinks? How often do you buy them?
2. What types of foods or drinks do you buy specifically for your youngest child (NAME)? What are your reasons for buying these foods or drinks? How often do you buy them?
3. If a food you usually buy is hard to find in the market, what do you do?

Probes:

- - - If it is unavailable, how do you decide what to buy instead?

1. What are some beliefs in your community about specific types of food?

Probes:

What foods are considered good? Why?

What foods are considered bad? Why?

1. How do positive beliefs about foods affect your decision to buy them? How do negative beliefs about foods affect your decision to buy them?
2. How important is how much your family likes or enjoys eating a food when you decide whether to buy it? How important is how much you like or enjoy eating a food when you decide whether to buy it?
3. How does cost affect what foods you buy for your family?

Probes:

- - - What food items will you still buy even if they are more expensive?
    - What food items will you NOT buy if they are too expensive?

1. What role does the amount of time you have available for preparing food play in what foods you decide to buy?

PILE SORT

1. Please sort the following items into three piles in terms of how you feel they influence your food and drink choices, starting with the items that never influence your food choices.

***ASK PARTICIPANTS TO SORT ITEMS IN THIS ORDER***

Pile 1: Never influence Pile 2: Always influence Pile 3: Sometimes influence

Of the following items, which ones do you think never influence your food and drink choices?

*Allow participant to put those items (if any) into a pile.*

Of those items that remain, which ones do you think always influence your food and drink choices?

*Allow participant to put those items (if any) into a pile.*

So of the remaining items, you think these sometimes influence your food and drink choices?

| Hunger/Appetite | Cooking skills or your ability to cook specific types of food |
| --- | --- |
| Taste | Health: when you’re feeling well you eat ___, when you’re unwell you eat ___ ___ |
| Food safety | Cost of food |
| Marketing/Advertisements | Seasonal availability |
| Mood/Cravings | Time available for food preparation |
| Healthiness or nutritional value of food | Attitudes/Beliefs about certain foods (including cultural beliefs/traditions) |

CLOSING QUESTION

1. Thank you so much for your time today and telling me about how you buy food for you and your family. Before we end our time together, is there anything else you’d like to share about your food purchasing habits or food preferences?
