# Supplementary material for: Food purchasing decisions of Malawian mothers with young children in households experiencing the nutrition transition
Source: Appetite. 2021 Jan 1;156:104855. doi: 10.1016/j.appet.2020.104855 (PMC7677890; doi:10.1016/j.appet.2020.104855)
Supplement: Multimedia component 6 [file mmc6.docx]

**HOUSEHOLD FOOD LOG**

Instructions: Ask the participants to list foods or ask to see foods currently available in the household and ask for other information in the table below.

Participant ID: ________________________ Date: ___________________ Name of Interviewer: __________________________

|  | **Food** | **Quantity**  **(include units)** | **Purchased or Self-produced** | **If purchased, how long ago?**  **(# days, wks, months)** | **If purchased, how often do you buy it?** | **If self-produced, how often do you need to buy it?** | **Value in Kwacha** |
| --- | --- | --- | --- | --- | --- | --- | --- |
| 1 |  |  |  |  |  |  |  |
| 2 |  |  |  |  |  |  |  |
| 3 |  |  |  |  |  |  |  |
| 4 |  |  |  |  |  |  |  |
| 5 |  |  |  |  |  |  |  |
| 6 |  |  |  |  |  |  |  |
| 7 |  |  |  |  |  |  |  |
| 8 |  |  |  |  |  |  |  |
| 9 |  |  |  |  |  |  |  |
| 10 |  |  |  |  |  |  |  |
| 11 |  |  |  |  |  |  |  |
| 12 |  |  |  |  |  |  |  |

|  | **Food** | **Quantity**  **(include units)** | **Purchased or Self-produced** | **If purchased, how long ago**  **(days, wks, months)** | **If purchased, how often do you buy it?** | **If self-produced, how often do you need to buy it?** | **Value in Kwacha** |
| --- | --- | --- | --- | --- | --- | --- | --- |
| 13 |  |  |  |  |  |  |  |
| 14 |  |  |  |  |  |  |  |
| 15 |  |  |  |  |  |  |  |
| 16 |  |  |  |  |  |  |  |
| 17 |  |  |  |  |  |  |  |
| 18 |  |  |  |  |  |  |  |
| 19 |  |  |  |  |  |  |  |
| 20 |  |  |  |  |  |  |  |
| 21 |  |  |  |  |  |  |  |
| 22 |  |  |  |  |  |  |  |
| 23 |  |  |  |  |  |  |  |
| 24 |  |  |  |  |  |  |  |
| 25 |  |  |  |  |  |  |  |
