# Supplementary material for: Food purchasing decisions of Malawian mothers with young children in households experiencing the nutrition transition
Source: Appetite. 2021 Jan 1;156:104855. doi: 10.1016/j.appet.2020.104855 (PMC7677890; doi:10.1016/j.appet.2020.104855)
Supplement: Multimedia component 7 [file mmc7.docx]

**Market Trip In-Depth Interview Guide**

Thank you for letting me join you on your trip to buy food for you and your family. I’d now like to ask you some questions about your food purchasing in general as well as some specific questions about today’s purchases.

OPENING QUESTIONS

1. In general, when you go to buy food for your family, what are the main factors that influence where you buy the food?
2. In general, when you go out to buy food for your family, what are the main factors that influence what you buy?
   1. Probes:
      1. What is the MOST important factor when you make food buying decisions?
      2. What is the LEAST important factor when you make food buying decisions?
3. How does the market trip we just went on compare to your usual trips to buy food?
   1. Probes:
      1. Were the places you bought food different today? In what way?
      2. Were the number or types of food your bought different? In what way?

SPECIFIC FOODS & LOCATIONS

1. Today you bought *(insert specific type of food)*. What was it about that particular food that led you to buy it? Can you tell me more about what you were thinking when you decided to buy it?
   1. Probes:
      1. How often do you buy this food?
      2. How much do you usually buy? What are the reasons for that?
      3. **If applicable:** Earlier, I heard you say *(insert something the woman said while choosing or purchasing)* about *(food item)*. Can you tell me more about that?
      4. **If applicable:** Earlier, I noticed *(insert observation from when woman was choosing or purchasing food item)*. What was going through your mind when that happened?
   2. **Ask these questions for all the foods (and drinks) she bought.**
2. Today we went to *(insert type of location – e.g., market, shop, or street vendor).*Can you tell me more about why you decided to go there to buy (some of) your food?
   1. Probes:
      1. Do you always go to that same location? Why?
   2. **Ask these questions for all the locations she went to buy food and beverages.**

SPECIFIC FACTORS

1. What types of foods or drinks do you buy specifically for your youngest child (NAME)? What are your reasons for buying these foods or drinks? How often do you buy them?
2. (Food Availability) If a food you usually buy is hard to find in the market, what do you do?
   1. Probes:
      1. If it is unavailable, how do you decide what to buy instead?
3. (Cultural beliefs / Social standing) If people in the community have certain beliefs (good or bad) about a specific type of food, how does that affect your decision of whether or not to buy it?
   1. Probes:
      1. What are foods are considered good?
      2. What are the foods are considered bad?
4. (Taste preferences) How important is how much your family likes or enjoys eating a food when you decide whether to buy it? How important is how much you like or enjoy eating a food when you decide whether to buy it?
5. (Cost) How does cost affect what foods you buy for your family?
   1. Probes:
      1. What food items will you still buy even if they are more expensive?
      2. What food items will you NOT buy if they are too expensive?
6. (Time constraints related to meal preparation / convenience)What role does the amount of time you have available for preparing food play in what foods you decide to buy?

PILE SORT

1. Please sort the following items into three piles in terms of how you feel they influence your food and drink choices, starting with the items that never influence your food choices.

***ASK PARTICIPANTS TO SORT ITEMS IN THIS ORDER***

Pile 1: Never influence Pile 2: Always influence Pile 3: Sometimes influence

Of the following items, which ones do you think never influence your food and drink choices?

*Allow participant to put those items (if any) into a pile.*

Of those items that remain, which ones do you think always influence your food and drink choices?

*Allow participant to put those items (if any) into a pile.*

So of the remaining items, you think these sometimes influence your food and drink choices?

| Hunger/Appetite | Cooking skills or your ability to cook specific types of food |
| --- | --- |
| Taste | Health: when you’re feeling well you eat ___, when you’re unwell you eat ___ |
| Food safety | Cost of food |
| Marketing/Advertisements | Seasonal availability |
| Mood/Cravings | Time available for food preparation |
| Healthiness or nutritional value of food | Attitudes/Beliefs about certain foods (including cultural beliefs/traditions) |

CLOSING QUESTION

1. Thank you so much for your time today and telling me about how you buy food for you and your family. Before we end our time together, is there anything else you’d like to share about today’s market trip that we didn’t already discuss?
