# Supplementary material for: Food purchasing decisions of Malawian mothers with young children in households experiencing the nutrition transition
Source: Appetite. 2021 Jan 1;156:104855. doi: 10.1016/j.appet.2020.104855 (PMC7677890; doi:10.1016/j.appet.2020.104855)
Supplement: Multimedia component 8 [file mmc8.docx]

**MARKET TRIP OBSERVATION**

Participant ID: ________________________ Date: ___________________

Name of Interviewer: __________________________

____________________________________________________________________________

|  |  |  |  |  | **OBSERVABLE FACTORS**  *Place a tick mark in the box if it appeared to play a role in the woman purchasing or not purchasing the item.* | | | |
| --- | --- | --- | --- | --- | --- | --- | --- | --- |
| **Food** | **Purchased?**  **Yes>**Quantity  **No** > Factors | **Quantity** *(If purchased)* | **Location of Purchase** | **Total Cost** | **Freshness** | **Availability** | **Price** | **Other**  *Write factor* |
|  |  |  |  |  |  |  |  |  |
|  |  |  |  |  |  |  |  |  |
|  |  |  |  |  |  |  |  |  |
|  |  |  |  |  |  |  |  |  |
|  |  |  |  |  |  |  |  |  |
|  |  |  |  |  |  |  |  |  |
|  |  |  |  |  |  |  |  |  |
|  |  |  |  |  |  |  |  |  |
|  |  |  |  |  |  |  |  |  |
|  |  |  |  |  |  |  |  |  |
|  |  |  |  |  |  |  |  |  |
